# Two new species of the leafhopper genus *Mitjaevia* Dworakowska from China (Hemiptera, Cicadellidae, Typhlocybinae)

**DOI:** 10.3897/zookeys.964.48655

**Published:** 2020-08-27

**Authors:** Xiaoxiao Chen, Yuehua Song

**Affiliations:** 1 School of Karst Science, Guizhou Normal University / State Key Laboratory Cultivation Base for Guizhou Karst Mountain Ecology Environment of China, Guiyang, Guizhou 550001, China Guizhou Normal University Guiyang China

**Keywords:** Checklist, distribution, Homoptera, identification key, morphology, taxonomy

## Abstract

In the present paper, two new species of the leafhopper genus *Mitjaevia* Dworakowska, 1970 from Guizhou Province China are described and illustrated, i.e., *Mitjaevia shibingensis***sp. nov.** and *Mitjaevia dworakowskae***sp. nov.** A checklist to species of the genus and a key to distinguish the Chinese species of the genus are given and the female valvulae are described and figured for the first time.

## Introduction

The leafhopper genus *Mitjaevia* Dworakowska, 1970 belongs to the tribe Erythroneurini of Typhlocybinae, with *Erythroneura amseli* Dlabola, 1961 as its type species; seventeen species are known, seven from China (see Checklist). Two new species from Guizhou Province, China are described and illustrated in this paper together with a checklist and key to species from China.

## Materials and methods

Specimens for this study were collected by sweep-net. Morphological terminology used follows Dietrich (2005) and Song and Li (2013) and observations and drawings were made using Olympus SZX16 and BX53 microscopes. Habitus photos were taken using a KEYENCE VHX-5000 digital microscope. Body measurements are from the apex of the vertex to the tip of the forewing. All specimens examined are deposited in the collection of the School of Karst Science, Guizhou Normal University, China (**GZNU**).

## Taxonomy

### Mitjaevia

Taxon classificationAnimaliaHemipteraCicadellidae

Dworakowska, 1970

CB58CCA0-E6C1-5EC1-A5E5-85E704CD6B6E

Mitjaevia Dworakowska, 1970: 763.

#### Type species.

*Erythroneura amseli* Dlabola, 1961, by original designation.

#### Diagnosis.

Species with distinctive dark brown markings; head distinctly narrower than pronotum; male pygofer with simple dorsal appendage and sometimes ventral appendage; subgenital plate with a series of lateral peg-like setae basally or subbasally and a few macrosetae centrally at midlength; style elongate with subapical extension and variably developed lateral lobe; aedeagus with shaft cylindrical or laterally compressed, with or without processes, with ventral gonopore, basal apodeme variably developed and preatrium distinct.

#### Distribution.

Palaearctic and Oriental Regions.

#### Remarks.

Dworakowska (1970: 763–765) gave a detailed description of this genus based on the three included species known at that time; based on subsequently included species a modified description was given by Song et al. (2011: 26–27) and Dmitriev (2020). In addition, the female valvulae are described and figured here for the first time. Although a diagnosis is given above, clearly further studies are needed to elucidate fully the diagnostic characters of the genus and to test if the genus is monophyletic in the light of the observed variation in male genitalia between species.

##### Checklist to species of the genus *Mitjaevia*

1 *Mitjaevia amseli* (Dlabola, 1961: 297, figs 137–141, *Erythroneura*. Uzbekistan); Dlabola 1964: 248, Afghanistan; Dworakowska 1970: 765, figs 33–44, transferred to *Mitjaevia*. Kazakhstan; Korolevskaya, 1976: 42–43, figs 7, 8.

2 *Mitjaevia atropictila* (Ahmed, 1970a: 35; fig. 5: A–F, *Erythroneura*. Pakistan); Sharma 1984: 33, figs 19–29, transferred to *Mitjaevia*. India.

3 *Mitjaevia aurantiaca* (Mitjaev, 1969: 1045; figs 1, 2, *Erythroneura*. Kazakhstan); Dworakowska 1970: 765, transferred to *Mitjaevia*; Korolevskaya 1976: 42, figs 9, 10.

4 *Mitjaevia aurea* Dworakowska, 1994: 118; figs 407–414. India.

5 *Mitjaevia bibichanae* (Dlabola, 1961: 296, figs 131–135, *Erythroneura*. Uzbekistan); Korolevskaya 1976: 43–44, figs 11–13, transferred to *Mitjaevia*. Tadzhikistan.

6 *Mitjaevia callosa* Dworakowska, 1980: 179; figs 263–272. India.

7 *Mitjaevia diana* (Distant, 1918: 100, *Typhlocyba*. India); Dworakowska 1970: 765; 1980: 179, figs 252–262, transferred to *Mitjaevia*. India, Kazakhstan.

8 *Mitjaevia elegantula* Dworakowska, 1994: 119; figs 415–425. India.

9 *Mitjaevia korolevskayae* Dworakowska, 1979: 44; figs 349–358. Vietnam.

10 *Mitjaevia maculata* (Ahmed, 1970b: 175; fig. 6: A–H, *Helionidia*. Pakistan); Dworakowska and Viraktamath 1975: 529, transferred to *Mitjaevia*. India.

11 *Mitjaevia nanaoensis* Chiang & Knight, 1990: 223; fig. 18: 1–7. China.

12 *Mitjaevia narzikulovi* Korolevskaya, 1976: 43; figs 1–6. Tadzhikistan.

13 *Mitjaevia notata* (Ahmed & Khokhar, 1971: 70; fig. 4a–f, *Helionidia*. Pakistan); Dworakowska 1980: 179, transferred to *Mitjaevia*. India.

14 *Mitjaevia protuberanta* Song, Li & Xiong, 2011: 27; figs 1–10. China.

15 *Mitjaevia shibingensis* sp. nov. China.

16 *Mitjaevia sikkimensis* Dworakowska, 1994: 119; figs 426–434. India.

17 *Mitjaevia dworakowskae* sp. nov. China.

18 *Mitjaevia tappana* Chiang & Knight, 1990: 224; fig. 19: 1–7. China.

19 *Mitjaevia wangwushana* Song, Li & Xiong, 2011: 29; figs 11–19. China.

### Key to species of *Mitjaevia* from China (males)

**Table:** 

1	Aedeagus with process	**2**
–	Aedeagus without process	**3**
2	Processes arising from aedeagal shaft subbasally (Figs 42, 43)	***M. protuberanta***
–	Processes arising from aedeagal shaft subapically (Figs 44, 45)	***M. wangwushana***
3	Aedeagus with shaft cylindrical, evenly tapered from base to apex (Figs 20, 34)	**4**
–	Aedeagus with shaft laterally compressed, abruptly tapered subapically to apex (Figs 45, 49)	**5**
4	Style lateral lobe small (Fig. 18); aedeagal shaft tapered to narrowly rounded apex in lateral view (Fig. 20)	***M. shibingensis* sp. nov.**
–	Style lateral lobe large (Fig. 31); aedeagal shaft tapered to acute apex in lateral view (Fig. 34)	***M. dworakowskae* sp. nov.**
5	Subgenital plate with few long macrosetae; aedeagus as in Figs 48, 49	***M. nanaoensis***
–	Subgenital plate with several long macrosetae; aedeagus as in Figs 46, 47	***M. tappana***

### Mitjaevia shibingensis sp. nov.

Taxon classificationAnimaliaHemipteraCicadellidae

EF29F1BC-0D8F-5681-82B6-E2722E65174B

http://zoobank.org/A8734F83-DBCD-4741-92C5-F2CF7AA083AC

Figs 1–7 , 15–27

#### Description.

**Figures 1–14.**
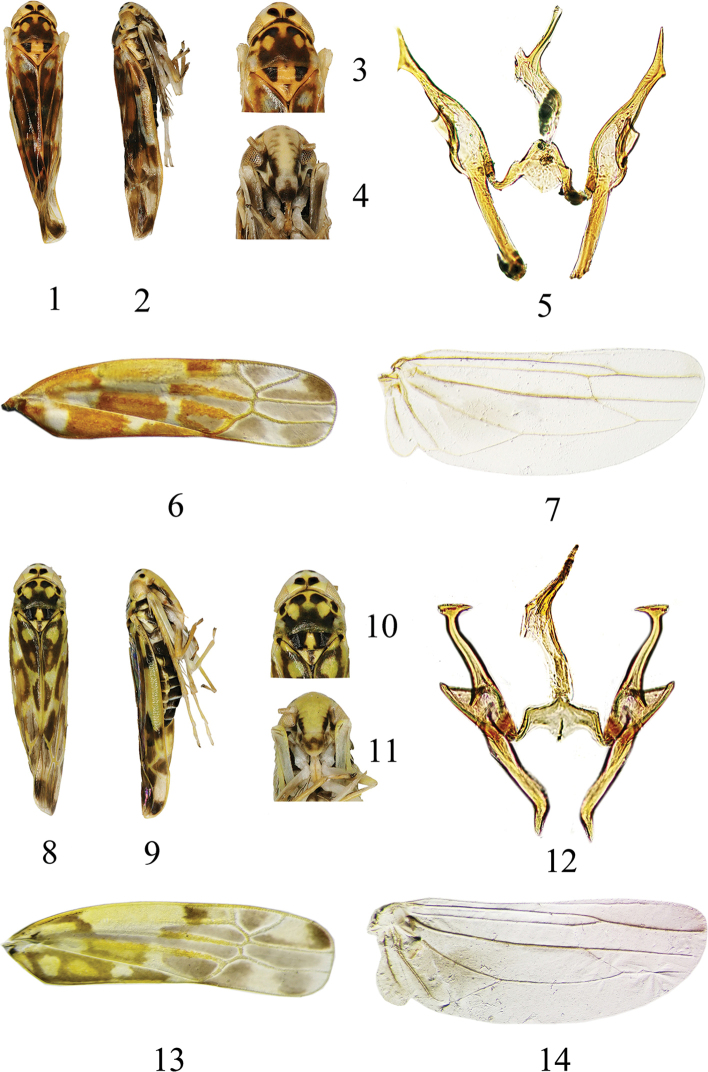
Species of *Mitjaevia***1–7***Mitjaevia shibingensis* sp. nov. **1** habitus, dorsal view **2** habitus, lateral view **3** head and thorax, dorsal view **4** face **5** style and connective, ventral view, aedeagus lateral view **6** forewing **7** hindwing **8–14***Mitjaevia dworakowskae* sp. nov. **8** habitus, dorsal view **9** habitus, lateral view **10** head and thorax, dorsal view **11** face **12** style and connective, ventral view, aedeagus lateral view **13** forewing **14** hindwing.

Vertex pale yellow, with pair of small black apical spots and two irregular markings at sides of coronal suture (Figs 1, 3). Face pale brownish yellow, anteclypeus with apical half dark brown; frontoclypeus with brownish black patches at sides basally (Fig. 4). Pronotum mostly dark brown, with pair of symmetrical brownish yellow oval impressed patches medially, showing brownish yellow near anterior margin (Figs 1, 3). Scutellum orange yellow, with brown irregular elliptical spot at base medially (Figs 1, 3). Forewing with orangey and gray patches (Fig. 6).

**Figures 15–27.**
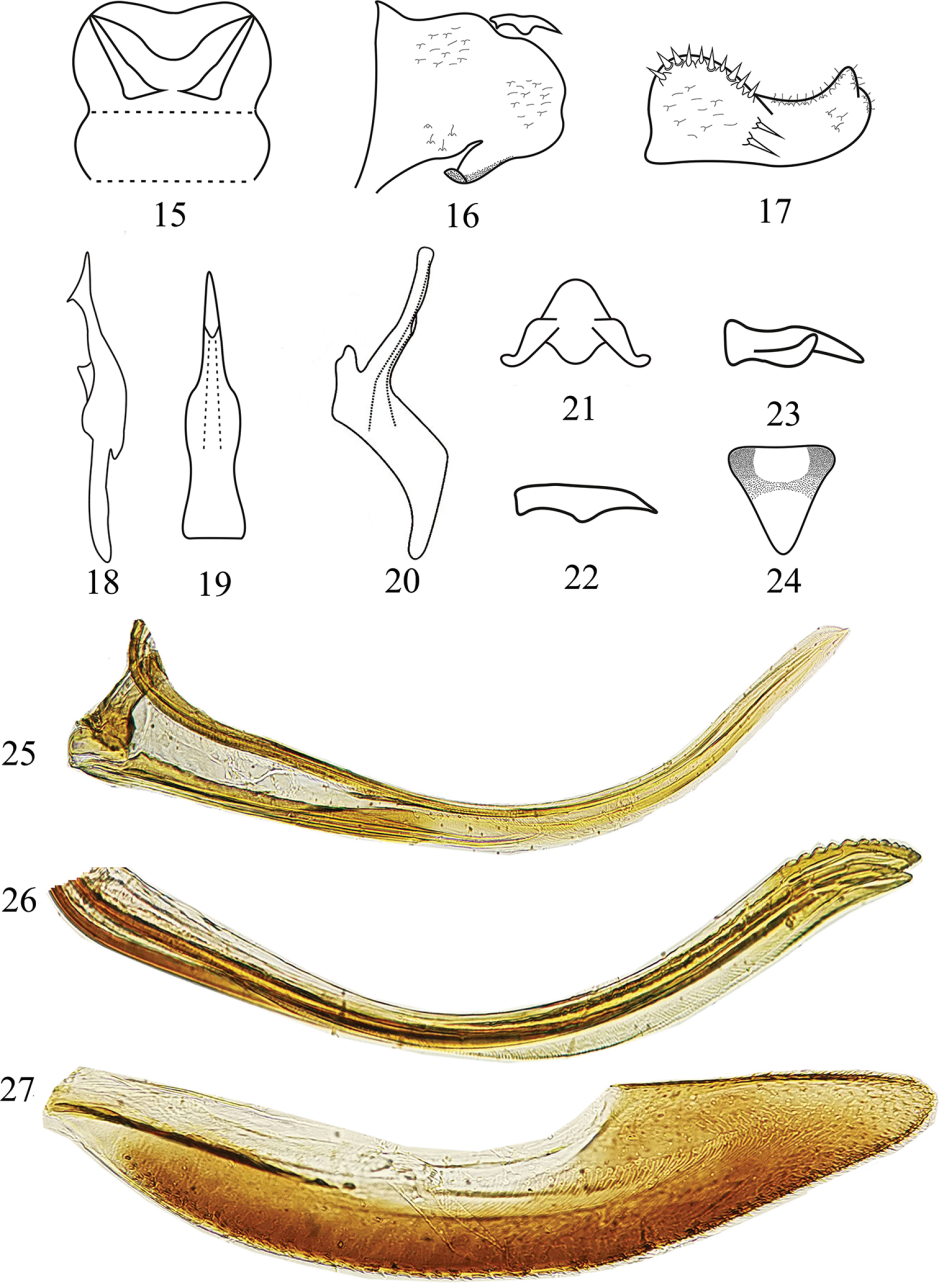
*Mitjaevia shibingensis* sp. nov. **15** abdominal apodemes **16** male pygofer, lateral view **17** subgenital plate, lateral view **18** style **19** aedeagus, ventral view **20** aedeagus, lateral view **21** connective **22** male pygofer dorsal appendage **23** male pygofer dorsal appendage **24** female 7^th^ sternite **25** valvula I **26** valvulae II **27** valvula III.

Abdominal apodemes small, not extended to hind margin of 3^rd^ sternite (Fig. 15). Male genitalia with subgenital plate relatively short, broadened basally, provided with two long macrosetae at midlength on lateral surface and numerous peg-like setae along dorsal margin basally to near midlength; several microsetae scattered on apical portion (Fig. 17). Style elongate, with subapical extension laterally, lateral lobe moderately large (Fig. 18). Aedeagal shaft narrow tapered to narrowly rounded apex in lateral view, gonopore arising near midlength on ventral surface; basal apodeme reduced; preatrium well developed (Figs 19, 20). Connective moderately broadly Y-shaped, central lobe well developed (Fig. 21). Female 7^th^ sternite as in Fig. 24. Valvula I elongate, curved dorsad and evenly tapered from base to apex, finely strigate along dorsal margin of apical 1/5 (Fig. 25). Valvulae II elongate, slightly expanded blade-like to near apex, thereafter tapered to down-turned apex, with few dorsal roundish teeth distally on right branch (Fig. 26). Valvula III tapered distally to narrowly rounded apex (Fig. 27).

#### Measurement.

Body length, males 2.6–2.8 mm, females 2.7–2.8 mm.

#### Specimen examined.

***Holotype*** ♂: China, Guizhou Prov., Shibing, 27 V 2019, coll. Zhouwei Yuan, Chao Tan and Xiaowei Yuan. ***Paratypes***: 14♂♂, 55♀♀, same data as holotype.

#### Remarks.

This species has a similar shaped aedeagus to *M. korolevskayae* but the style has a preapical extension (“heel”) and a smaller lateral lobe.

#### Etymology.

The new species is named after its type locality: “Shibing”, Guizhou Province.

### Mitjaevia dworakowskae sp. nov.

Taxon classificationAnimaliaHemipteraCicadellidae

9F1FEF78-030E-5541-9588-2936A2917C06

http://zoobank.org/AE8B70FC-D3C5-4F7E-ACA6-1B23044AAB49

Figs 8–14 , 28–41

#### Description.

Vertex light yellow, with two pairs of irregular black preapical spots distributed symmetrically (Figs 8, 10). Face milky yellow, anteclypeus with central area brownish; frontoclypeus with brownish black patches at sides basally (Fig. 11). Pronotum mostly black, with pair of symmetrical pale-yellow oval impressed patches medially, also showing pale yellow near anterior margin (Figs 8, 10). Scutellum milky yellow, with longitudinal black stripe between scutellar suture and apex (Figs 8, 10). Forewing with brown and brownish yellow patches (Fig. 13).

**Figures 28–41.**
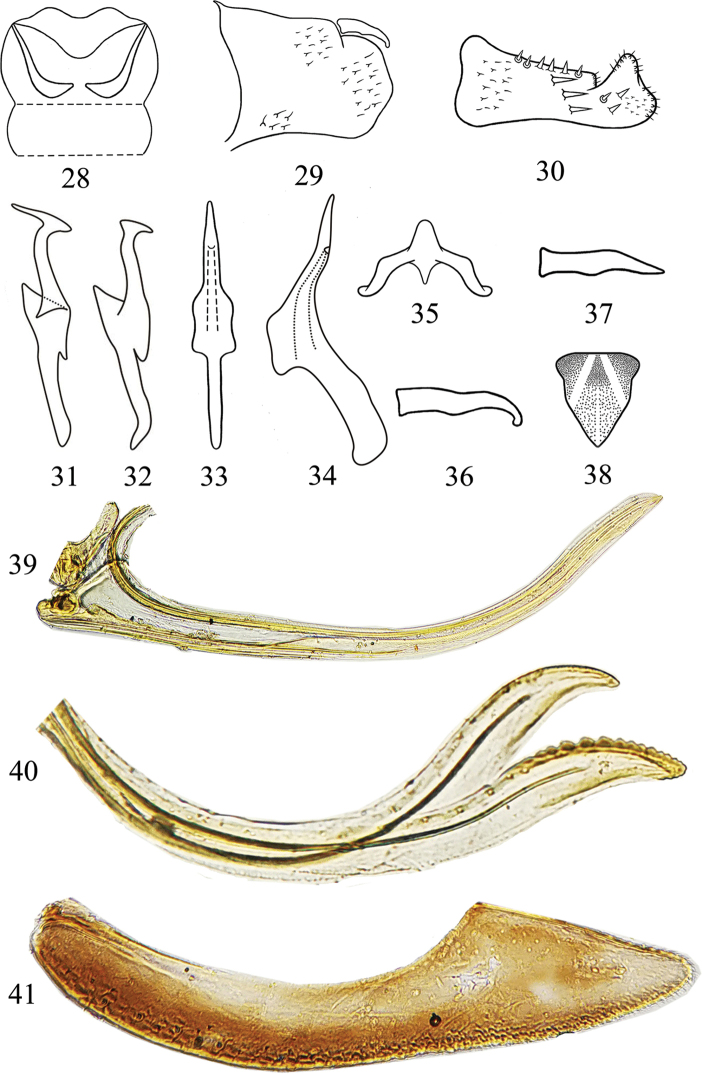
*Mitjaevia dworakowskae* sp. nov. **28** abdominal apodemes **29** male pygofer lobe, lateral view **30** subgenital plate **31** style **32** style **33** aedeagus, ventral view **34** aedeagus, lateral view **35** connective **36** pygofer dorsal appendage **37** pygofer dorsal appendage **38** female 7^th^ sternite **39** valvula I **40** valvulae II **41** valvula III.

Abdominal apodemes small, not extended beyond hind margin of 3^rd^ sternite (Fig. 28). Male genitalia with subgenital plate laterally with 3 macrosetae at midlength and three more distal shorter macrosetae, dorsal peg-like setae restricted to central part (Fig. 30). Style elongate with preapical extension on inner surface, lateral lobe large (Figs 31, 32). Aedeagal shaft narrow slightly sinuate and tapered to acute apex in lateral view with gonopore arising near midlength of ventral surface; basal apodeme reduced (Figs 33, 34). Connective broadly Y-shaped, central lobe slender (Fig. 35). Female 7^th^ as in Fig. 38. Valvulae as in previous species (Figs 39–41).

#### Measurement.

Body length, males 2.3–2.4 mm, females 2.4–2.5 mm.

#### Specimen examined.

***Holotype*** ♂: China, Guizhou Prov., Shibing, 27.V.2019, coll. Zhouwei Yuan, Chao Tan and Xiaowei Yuan. ***Paratypes***: 14♂♂, 19♀♀, same data as holotype.

#### Remarks.

This species can be distinguished by the narrow and slightly sinuate aedeagal shaft in lateral view and the style with a subapical extension on the inner surface with a greatly enlarged lateral lobe.

#### Etymology.

This species is named for Dr Irina Dworakowska in recognition of her immense contribution to taxonomy of World Typhlocybinae.

**Figures 42–49.**
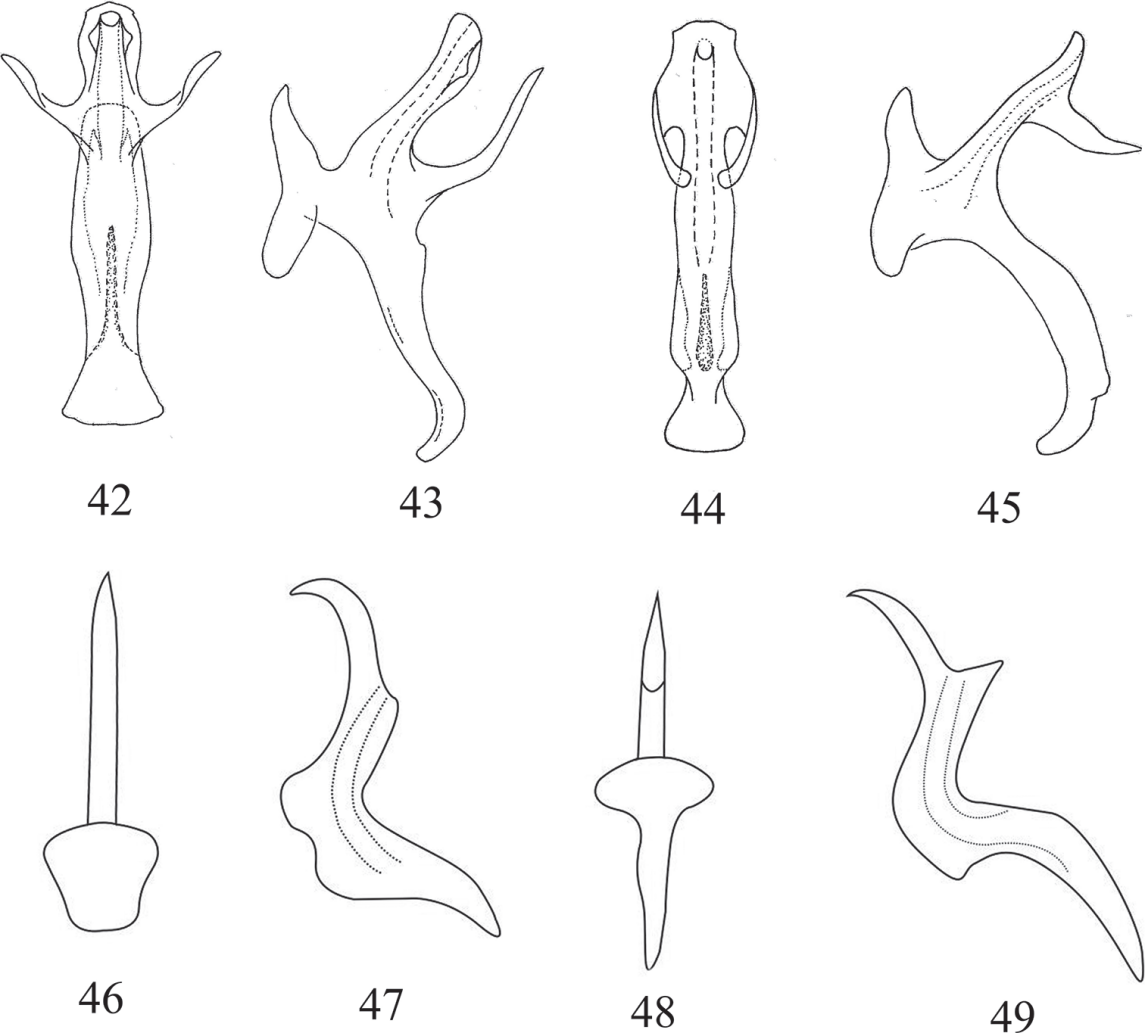
Species of Chinese *Mitjaevia***42, 43***M. protuberanta* Song, Li & Xiong **42** aedeagus, ventral view **43** aedeagus, lateral view **44, 45***M. wangwushana* Song, Li & Xiong **44** aedeagus, ventral view **45** aedeagus, lateral view **46, 47***M. tappana* Chiang & Knight **46** aedeagus, ventral view **47** aedeagus, lateral view **48, 49***M. nanaoensis* Chiang & Knight **48** aedeagus, ventral view **49** aedeagus, lateral view (Figs **42–45**, from original; Figs **46–49**, redrawn from Chiang and Knight 1990).

## Supplementary Material

XML Treatment for Mitjaevia

XML Treatment for Mitjaevia shibingensis

XML Treatment for Mitjaevia dworakowskae
